# Acute Aortic Dissection Presenting with a Headache: An Easily Missed Life-threatening Emergency

**DOI:** 10.7759/cureus.3531

**Published:** 2018-10-31

**Authors:** Johnny Chahine, Bicky Thapa, Rama D Gajulapalli, Amer Kadri

**Affiliations:** 1 Internal Medicine, Cleveland Clinic - Fairview Hospital, Cleveland, USA

**Keywords:** aortic dissection, headache

## Abstract

Acute aortic dissection is a deadly disease that should be recognized promptly. We report an exceptional case of a 44-year-old African American female who presented with a rapidly progressing severe frontal headache. Initial computed tomography of the brain was negative. The following day, she developed uncontrolled hypertension and worsening headache. Magnetic resonance imaging of the brain was therefore done and showed evidence of acute/subacute ischemic infarcts. The patient was managed as having an ischemic stroke. For that reason, an echocardiogram was done the next day that showed a dilated aortic root and moderate-to-severe aortic regurgitation. This was followed by a computed tomography angiography which showed ascending aortic dissection involving bilateral common carotid arteries. After an urgent surgical intervention, the patient recovered without any sequelae. Patients with an acute dissection can present with atypical clinical features, such as an isolated rapidly progressive headache, which might delay the diagnosis and jeopardize their lives. Hence, high-risk patients with rapidly progressive unexplained severe headaches should be considered for imaging of the aorta.

## Introduction

Acute aortic dissection is a catastrophic medical emergency that can affect different parts of the thoracic aorta [1]. Predisposing conditions, such as high blood pressure and connective tissue diseases (like Marfan syndrome), might degenerate the aortic wall. Other associated conditions include atherosclerosis, bicuspid or surgically replaced aortic valve, cocaine use disorder, and Turner syndrome [1-4]. We report a rare case of rapidly progressing severe headache as the sole presenting symptom of this life-threatening condition.

## Case presentation

A 44-year-old African American lady with a past medical history of schizophrenia and hypertension presented to the emergency department with a rapidly progressing severe throbbing frontal headache of 10 over 10 intensity. The headache started a few hours before presentation and was associated with generalized weakness, blurry vision, and dizziness. She denied nausea, vomiting, head trauma, any focal neurologic deficit (including loss of sensation or motor power, gait disturbance, dysarthria, and dysphagia), loss of consciousness, chest pain, back pain, shortness of breath, palpitations, or any personal or family history of intracranial bleeding. She is a 30 pack/year smoker with no other history of substance use. Her blood pressure was 107/51 mmHg, her heart rate was 60 beats/min, her respiratory rate was 16 breaths/min, her temperature was 36.6°C, and her oxygen saturation was 95% on room air. On neurologic examination, her cranial nerves examination was insignificant, and there were no other focal neurologic deficits. Signs of meningeal irritation were absent, and papilledema was not seen on fundoscopy. The remainder of the examination was unremarkable. Her laboratory workup showed normal hemoglobin, platelets, white blood cells, renal function, hypokalemia of 2.7 mmol/L (normal: 3.5-5.0 mmol/L), and hypoalbuminemia of 2.9 g/dL (normal: 3.4-5 g/dL) (Table 1).

**Table 1:** Laboratory values on admission

Laboratory parameter	Value	Reference range
Hemoglobin	12.8 g/dL	12.0-15.0 g/dL
Platelets count	282 k/uL	140-440 k/uL
White blood cells	9.7 k/uL	3.9-11.0 k/uL
Sodium	144 mmol/L	135 - 146 mmol/L
Potassium	2.7 mmol/L	3.5 - 5.0 mmol/L
Creatinine	0.83 mg/dL	0.70 - 1.40 mg/dL
Blood urea nitrogen	12 mg/dL	10 - 25 mg/dL
Total bilirubin	0.5 mg/dL	0.2-1.0 mg/dL
Aspartate aminotransferase	10 u/L	15-37 u/L
Alanine aminotransferase	10 u/L	12-78 u/L
Albumin	2.9 g/dL	3.4-5 g/dL

**Figure 1:**
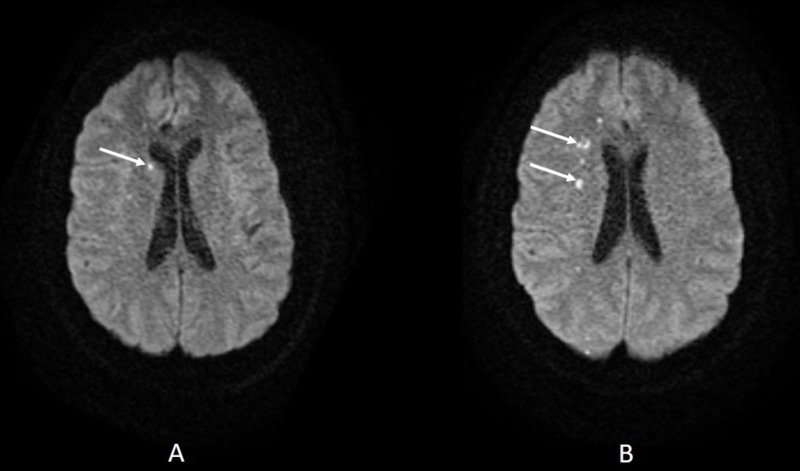
Diffusion-weighted magnetic resonance imaging of the brain showing punctate infarcts (white arrows).

A brain computed tomography (CT) scan was negative for intracranial bleeding. She was then admitted to the regular nursing floor with opioids as needed for her headache. The following day, her blood pressure became uncontrolled with values up to 200/95 mmHg, and she reported a worsening frontal headache. A magnetic resonance imaging (MRI) of the brain and its vessels was done that showed possible punctate lacunar infarcts without evidence of a vascular aneurysm, venous thrombosis, or a space-occupying lesion (Figure 1A).

The patient was treated as per the standard ischemic stroke protocol: aspirin and statin were initiated as well as nicardipine infusion as needed. The next day, she developed transient chest pain and sudden onset of left leg numbness. On physical examination, a new parasternal early diastolic murmur was found with no significant change in the neurologic examination. A repeat MRI of the brain showed scattered foci of restricted diffusion within the right frontal, parietal, and superior right occipital lobes compatible with an acute or early subacute infarction (Figure 1B).

**Figure 2:**
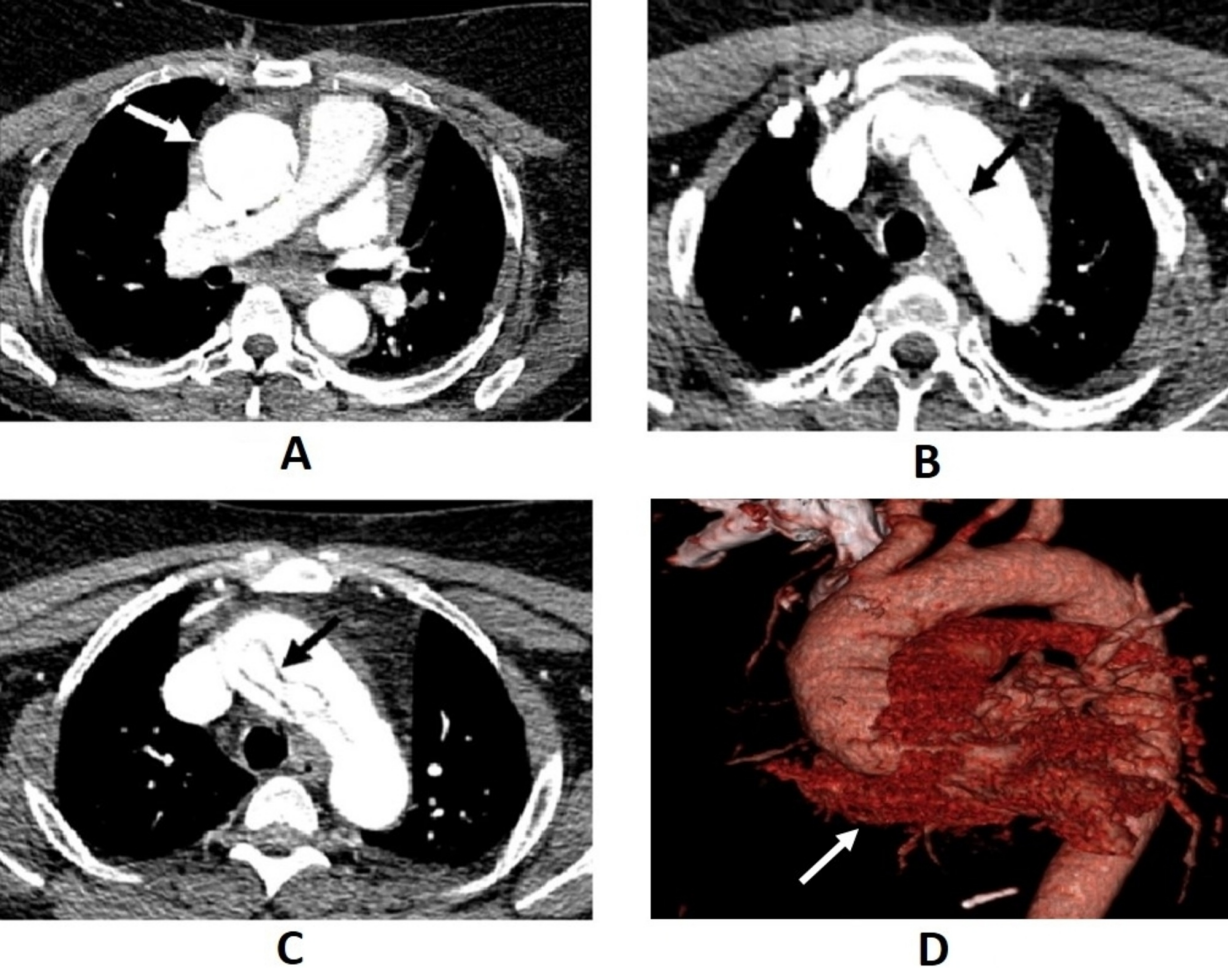
Computed tomography angiography showing (A) dilation of the ascending aorta with a diameter around 44 mm, (B and C) dissection of the ascending aorta, and (D) three-dimensional reconstruction of the aorta (with the false lumen in dark red).

As a part of the routine stroke/transient ischemic attack workup, transthoracic echocardiography was done, which showed the diameter of the aortic root to be 4.0 cm (normal: less than 2.1 cm) and moderate-to-severe aortic regurgitation. A CT angiography of the chest showed an ascending thoracic aortic dissection with extension into the origin of the left common carotid, right innominate, and right common carotid arteries with significant (90%) luminal narrowing of the proximal right common carotid artery (Figure 2).

Vascular surgery was emergently consulted, and the patient had a successful surgical repair with no significant complications.

## Discussion

Patients with a type A aortic dissection typically present with an abrupt onset of severe chest pain (78.9%). Less frequent presentations are abdominal pain, syncope, and ischemic neuropathy [1]. Isolated headache and cervicalgia at presentation are infrequent [5-8], as the involvement of the proximal common carotid artery is uncommon [5-9], and are generally seen in patients with internal carotid artery dissections [10]. On examination, aortic regurgitation murmur has only been found in 44% of patients with type A dissections, uncontrolled hypertension in 35%, pulse abnormality in 19%, and neurologic findings in about 6% of cases [1].

The diagnosis of an acute aortic dissection could be challenging as the symptoms simulate more common diseases [11]. In one study, 39% of the patients with acute aortic dissection got diagnosed after 24 hours of admission [12]. As a matter of fact, physicians have been reported to initially suspect acute aortic dissection in less than half of the patients who have it [2]. Widening of the mediastinum was only seen in 62% of cases and the findings on electrocardiography are nonspecific [1-2]. The sensitivity and specificity of CT angiography of the chest are close to 100% [13], and helical CT can even diagnose atypical subtypes of dissections and is useful for postoperative monitoring. Triple-rule-out CT angiography can be useful in cases of chest pain in the emergency department to rule out aortic disease, pulmonary embolism, and coronary artery disease; however, specific indications for this test are still unclear [14]. MRI is as accurate [13]; however, its use is limited to stable patients. In unstable patients, echocardiography is an alternative to CT. The sensitivity of transesophageal echocardiography is higher than a transthoracic one (more than 90% versus 59%), though the former has a slightly lower specificity (77% versus 83%, respectively) [15-16].

Type A aortic dissection is a surgical emergency [4-5], with inpatient mortality of 24% when a surgical approach is used as compared to 58% with medical treatment alone (p-value less than 0.0001) [17]. Promising results have been seen with endovascular repair of acute aortic dissections [18], but no solid recommendations for this approach have been approved yet.

Blood pressure control is crucial to prevent further shear of the aortic intima. Medications with negative inotropic activity (mainly beta blockers) are the first line, with target systolic blood pressure below 120 mmHg and heart rate below 60 beats/min. If that fails, vasodilators and angiotensin-converting enzyme inhibitors could be added [4].

## Conclusions

Type A aortic dissection is a life-threatening surgical emergency. Unfortunately, its diagnosis is sometimes challenging due to atypical presentations. In high-risk patients (like those with chronic hypertension and connective tissue disorders) reporting rapidly progressing severe headache—and after excluding an intracranial pathology—diagnostic imaging of the thoracic aorta should be considered. Type A aortic dissection has high mortality regardless of the therapeutic approach, but it can be lowered with early diagnosis and surgical intervention.
